# Recruitment of cortical silent responses by forskolin in the anterior cingulate cortex of adult mice

**DOI:** 10.1177/17448069241258110

**Published:** 2024-05-23

**Authors:** Yujie Ma, Jinjin Wan, Shun Hao, Qi-Yu Chen, Min Zhuo

**Affiliations:** 1 Oujiang Laboratory (Zhejiang Lab. for Regenerative Medicine, Vision and Brain Health), 26453 Wenzhou Medical University, Wenzhou, China; 2 Zhuomin Institute for Brain Research, Qingdao International Academician Park, Qingdao, China; 3 Department of Physiology, Faculty of Medicine, 7938 University of Toronto, Toronto, ON, Canada

**Keywords:** adenylyl cyclase, cyclic adenosine monophosphate, long-term potentiation, α-amino-3-hydroxy-5-methyl-4-isoxazole-propionicacid receptor, silent response

## Abstract

Recent studies using different experimental approaches demonstrate that silent synapses may exist in the adult cortex including the sensory cortex and anterior cingulate cortex (ACC). The postsynaptic form of long-term potentiation (LTP) in the ACC recruits some of these silent synapses and the activity of calcium-stimulated adenylyl cyclases (ACs) is required for such recruitment. It is unknown if the chemical activation of ACs may recruit silent synapses. In this study, we found that activation of ACs contributed to synaptic potentiation in the ACC of adult mice. Forskolin, a selective activator of ACs, recruited silent responses in the ACC of adult mice. The recruitment was long-lasting. Interestingly, the effect of forskolin was not universal, some silent synapses did not undergo potentiation or recruitment. These findings suggest that these adult cortical synapses are not homogenous. The application of a selective calcium-permeable AMPA receptor inhibitor 1-naphthyl acetyl spermine (NASPM) reversed the potentiation and the recruitment of silent responses, indicating that the AMPA receptor is required. Our results strongly suggest that the AC-dependent postsynaptic AMPA receptor contributes to the recruitment of silent responses at cortical LTP.

## Introduction

Long-term potentiation (LTP) is a key form of synaptic plasticity that is important for brain functions in physiological conditions and diseases in pathological conditions.^1–3^ Cumulative evidence demonstrates that LTP in the anterior cingulate cortex (ACC) is tightly related to chronic pain.^4,5^ In the ACC, two major forms of LTP have been reported: presynaptic and postsynaptic form of LTP (pre-LTP and post-LTP).^1,6^ Both forms of LTP in the ACC require activation of calcium-stimulated adenylyl cyclases (ACs) including adenylyl cyclases subtype 1 (AC1).^6–9^ While pre-LTP is likely due to the increase of glutamate release, post-LTP is mediated by postsynaptic modification of AMPA receptors or insertion of AMPA receptors into synapses.^1,10,11^ For example, genetic deletion of AC1 or pharmacological inhibition of AC1 using a selective inhibitor NB001 inhibited post-LTP induced by theta-burst stimulation (TBS) in the ACC.^12,13^

Silent synapses have been reported in different regions of the brain, such as the hippocampus, cortex, and spinal cord.^14–17^ However, most of these studies were recorded from young neurons.^14,15^ It has been suggested that silent synapses may not exist in adult synapses or are only expressed at a low level. A recent study in the adult sensory (visual) cortex by Vardalaki et al (2022) found that silent synapses can be detected at layer five pyramidal neurons in the primary visual cortex of adult mice and they also demonstrated that filopodia could as a structural basis for silent synapses.^18^ In our previous studies of adult ACC LTP, we have found that some silent responses in the ACC slices of adult mice can be recruited after TBS-induced LTP.^17,19,20^ Calcium-stimulated AC1 and calcium-permeable AMPA receptor subtype 1 are found to be important for this recruitment.^7,10^ However, there is no evidence that the chemical activation of ACs may recruit silent responses in the ACC of adult mice.

In the present study, we used the multielectrode 64-channel (MED64) recording system to investigate if forskolin, an activator of ACs, recruit silent responses in the ACC. We also wanted to examine if the recruited responses are mediated by calcium-permeable AMPA receptors (CP-AMPA receptors). We found that forskolin recruited silent responses in the ACC of adult mice, and these newly recruited responses were CP-AMPA receptor-dependent.

## Methods

### Animals

Adult male C57BL/6 mice (6–8 weeks old) were purchased from the Beijing Vital River Laboratory Animal Technology Co., Ltd. All mice were randomly housed in corncob-lined plastic cages under an artificial 12 h light/12 h dark cycle (lights on 9 a.m. to 9 p.m.) with enough food and water. Animal protocols were approved by the Ethics Committee of Oujiang Laboratory.

### Brain slice preparation

Coronal brain slices (300 μm) containing the ACC from C57BL/6 mice were prepared using standard methods.^21^ Briefly, mice were anesthetized with 1%–2% isoflurane and sacrificed by decapitation. The whole brain was quickly removed from the skull and submerged in the ice-cold oxygenated (95% O_2_ and 5% CO_2_) artificial cerebrospinal fluid (ACSF) containing (in mM) 124 NaCl, 2.5 KCl, 2 CaCl_2_, 2 MgSO_4_, 25 NaHCO_3_,1 NaH_2_PO_4_, and 10 glucose, pH 7.3–7.4. After cooling for a short time, the whole brain was trimmed for an appropriate part to glue onto the cutting staged tissue slicer of a Leica VT1200S Vibratome. Slices were transferred to a submerged recovery chamber with oxygenated (95% O_2_ and 5% CO_2_) ACSF at room temperature for at least 1 h.

### Preparation of the multielectrode array

A commercial MED64 recording system (Panasonic) was used for extracellular field potential recordings. The procedure for preparation of the MED64 probe (P515 A, Panasonic) used standard methods.^21^ The MED64 probe has an array of 64 planar microelectrodes, each arranged in an 8 × 8 pattern, with an interelectrode distance of 150 mm. Before use, the surface of the MED64 probe was treated with 0.1% polyethyleneimine (P-3143, Sigma-Aldrich) in 25 mmol/L borate buffer, pH 8.4, overnight at room temperature. Before using it in the experiments, the probe surface was rinsed at least three times with sterile distilled water.

### Field potential recording in adult ACC slices

**Figure 1.**
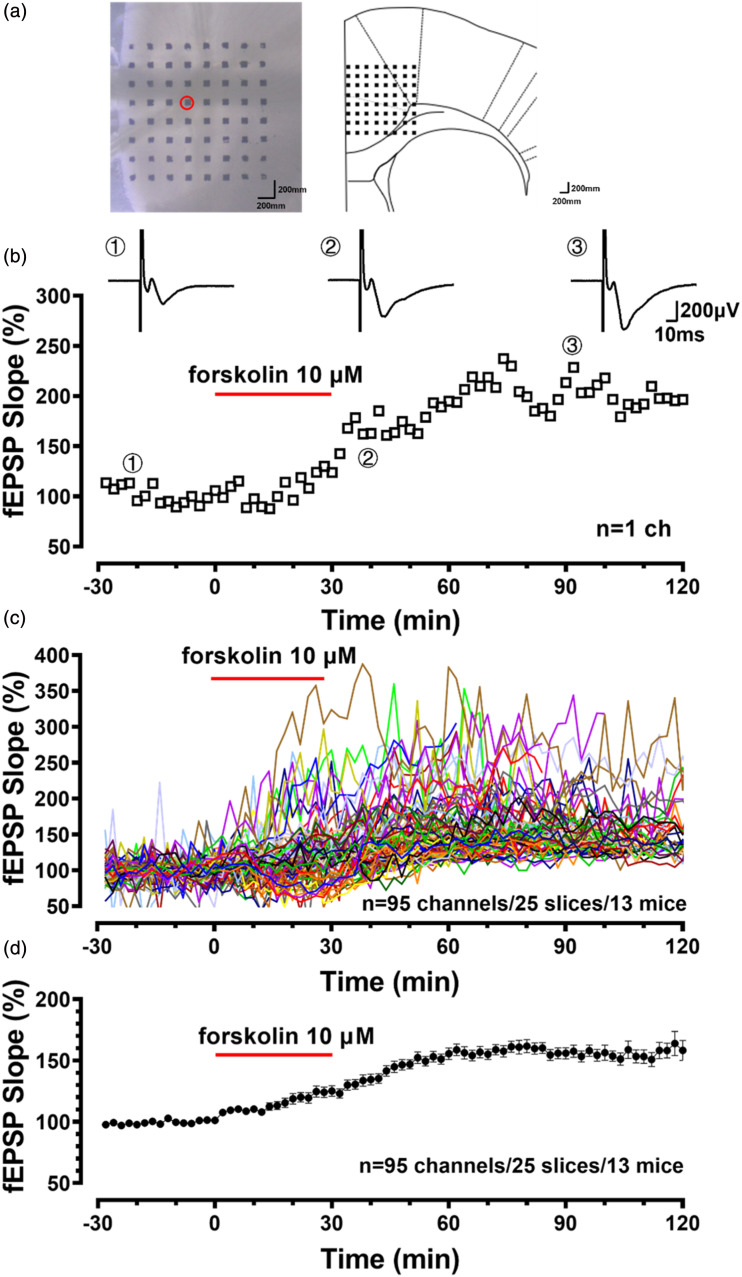
Forskolin-induced LTP of the fEPSPs in the ACC (a) Microphotograph and schematic diagram showed one example of fEPSP recording on the coronal slices of the ACC by using the MED64 system. A coronal brain slice containing the ACC was placed on a probe with 64 electrodes (MED-P515 A, 8 × 8 array). One channel of the probe (red circle) was selected as the stimulation site. The evoked field potentials in all the other 63 channels were recorded 30 min before and 90 min after theta-burst stimulation (TBS). (b) The fEPSP slope (bottom) and the potentiated samples (top) from one channel show that forskolin-induced the potentiation. (c), (d) Single and averaged fEPSP slopes of 95 channels with LTP after forskolin.

After incubation, one slice containing the ACC was transferred to the prepared MED64 probe and perfused with the oxygenated fresh ACSF at room temperature and maintained at a flow rate of 2 mL/min. The slice was positioned on the MED64 probe in such a way that the different layers of the ACC were entirely covered by the whole array of the electrodes shown in Figure 1(a), and then a fine-mesh anchor was placed on the slice to ensure its stabilization during the experiments. After at least a 1 h recovery period for the slices in the recording chamber, biphasic constant-current pulse stimulation (0.2 ms) was applied to the stimulation channel, and the intensity was adjusted so that a half-maximal field excitatory postsynaptic potential (fEPSP) was elicited in the channels closest to the stimulation site. The parameter of ‘slope’ indicated the average slope of each fop recorded by activated channels. Stable baseline responses were first recorded until the baseline response variation was 5% in most of the active channels within 1 h. Then, a bathing application of forskolin (10 μM) was given to induce LTP.^7,9^

### Data analysis

The data is presented as means ± standard error of the mean (SEM). Statistical comparisons between two groups were performed using two-tail paired Student’s t-test, one-way analysis of variance (ANOVA) to identify significant differences. In all cases, *p* < .05 was considered statistically significant.

## Results

### Forskolin-induced LTP of fEPSPs using MED64 in the ACC

In previous studies, by using dual whole-cell patch clamp recording^9,22^ and behavior^23^ methods, we found that ACs play a critical role in synaptic plasticity in the ACC. However, few studies have reported that the MED64 recording system was used to record cortical circuit responses. Here, we used the MED64 recording system to characterize the intracellular connections within the ACC in response to focal electrical stimulation. As shown in Figure 1(a), one channel in the deeper layer of the ACC (layer Ⅴ) was chosen as the site of stimulation, and the other 63 channels were used to measure the evoked responses. For example, Figure 1(b) shows the fEPSP slope (bottom) and the potentiated samples (top) from one channel evoked by forskolin. Within the 63 recorded channels, after bath application of forskolin (10 μM), LTP was induced in most of the recording channels with the maximum of the fEPSP slopes over 300 % (*n* = 95 channels/25 slices/13 mice; Figure 1(c)). Forskolin-induced LTP reached the peak about 30 min after washout and lasted for at least 1 h (156.9 ± 4.6% of the baseline, Figure 1(d)). These results are consistent with our previous work, indicating that the LTP is induced by forskolin using the MED64 recording system.

### Forskolin-induced LTP did not appear in all the responsive channels in the ACC

**Figure 2.**
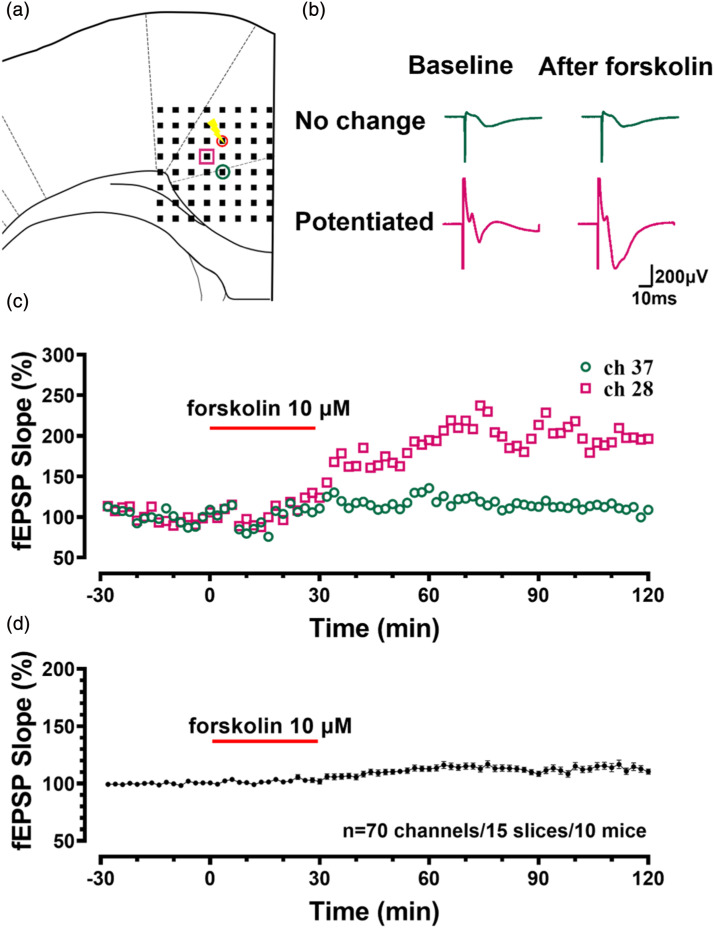
Not all the responsive channels of the evoked fEPSPs were potentiated by forskolin in the ACC (a) The schematic diagram showed one example of ACC slices containing channels with and without potentiation (the pink square indicates a responsive channel with potentiation, the green circle indicates a responsive channel without potentiation, and the red circle with the flashlight mark indicates a channel of the stimulation site). (b) sample traces of no change and potentiated fEPSPs before and after the application of forskolin in the same slice. (c) The fEPSPs slope of the no-change and potentiated channels in (a). (d) The summarized fEPSP slope shows all non-potentiated channels after forskolin.

We applied forskolin to investigate whether it could induce LTP in all the channels that have been activated by the electric stimulation in the ACC slices using the MED64 recording system. We found that not all channels responded to forskolin in the same brain slice. Figure 2(a) shows an example diagram of the stimulus and recording site. After the forskolin administration, the potentiated channel increased to 203.5% of baseline, while the channel without LTP increased by less than 120% (Figure 2(c)). Figure 2(b) shows sample traces of channels with and without LTP after the application of forskolin. The fEPSP slope of these 70 channels without LTP was 112.7 ± 2.2% of the baseline (*n* = 15 slices/10 mice, Figure 2(d)). In summary, forskolin-induced LTP does not occur at all recording sites in the ACC.

### Recruitment of synaptic responses in the ACC after applying forskolin

**Figure 3.**
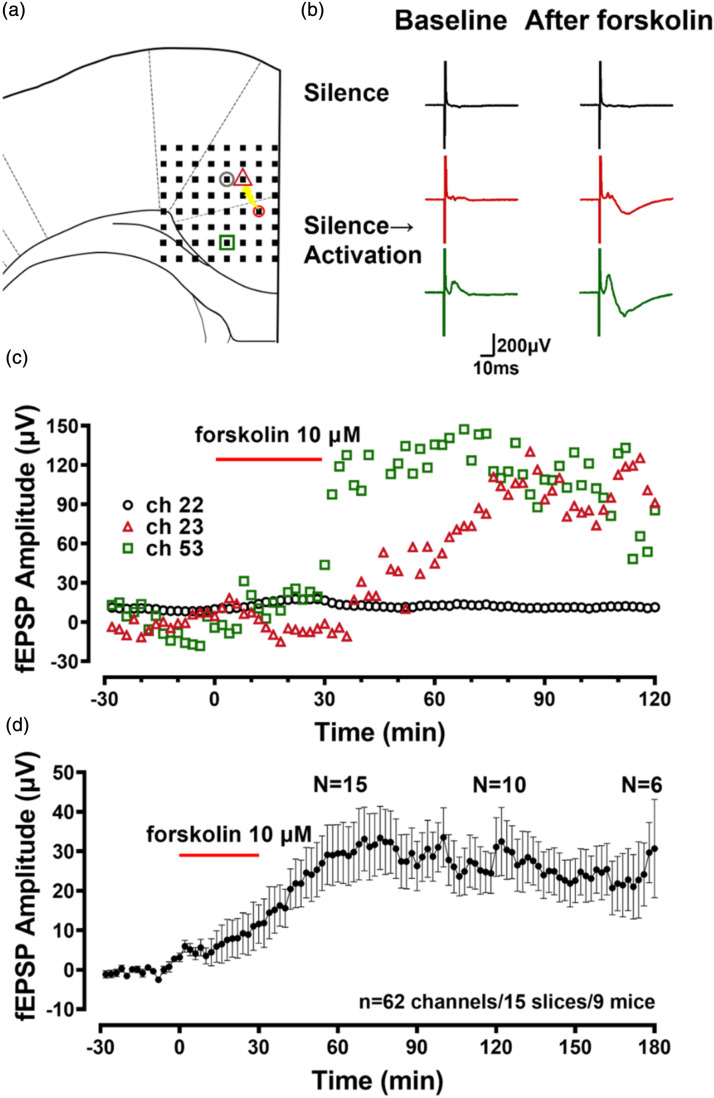
Recruited channels of the fEPSP recordings after forskolin induction in the ACC (a) A schematic diagram showed one example containing silent and silent-to-active channels for fEPSP recording in the ACC (the pink square indicates a responsive channel with potentiation, the green circle indicates a responsive channel without potentiation, the gray circle indicates a channel with recruited response, and the red circle with the flashlight mark indicates a channel that is the stimulation site). (b) The traces show that the channel has not been altered (top), and recruited channels from silent to active (bottom) after the application of forskolin. (c) The fEPSP amplitudes show the change of the amplitude of three channels before and after the forskolin application in (a). (d) The averaged amplitude of fEPSP from all recruited channels before and after the forskolin application in the ACC. N (above of curve) shows the number of slices at 60 min, 120 min, and 180 min after forskolin administration.

Just as the recruitment of silent responses induced by TBS in the ACC,^19,24^ forskolin also induced the recruitment of silent synaptic responses. The newly recruited channels are located at the edge of the originally active area (Figure 3(a)). There is a significant change in the response of these recruited channels from silencing to activation, and their amplitudes exceed 120 μA (Figure 3(b) and (c)). However, the increased amplitude in the above Figure was not seen in all channels. After forskolin perfusion, the amplitude of fEPSPs of recruited channels gradually increased (finally reached as large as 33.5 ± 7.5 μA) and maintained stable for 3 h (*n* = 62 channels/15 slices/9 mice, Figure 3(d)).

**Figure 4.**
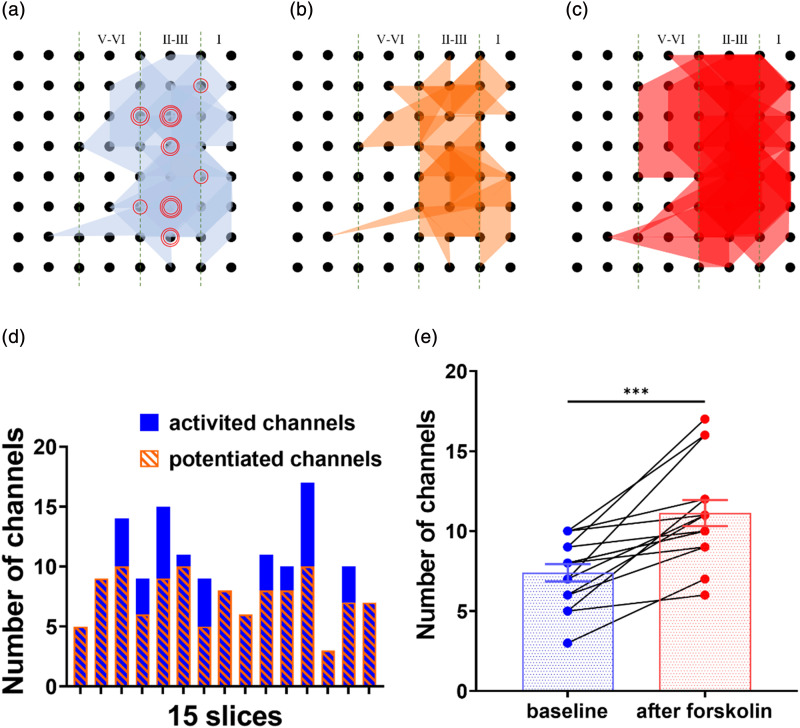
Recruited synaptic responses within the ACC after LTP induction (a) The superimposed polygonal diagram showed the distribution of activated channels with fEPSP in the baseline state (blue). The red circle indicates the stimulation site (*n* = 15 slice/9 mice). (b) The superimposed polygonal diagram showed the distribution of potentiated channels (orange) in activated channels after the application of forskolin. (c) The red polygonal diagram showed the enlarged area from the baseline state after the application of forskolin. (d) The number of activated channels (blue) of baseline and potentiated channels (orange shaded) after the application of forskolin. (e) The number of activated channels before and after forskolin-induced recruited responses. (Paired two-tailed t-test, t = 5.332, ***p < .001).

In the ACC, we found that channels with LTP, without LTP, and recruited channels after forskolin perfusion coexisted (Figure 4(a)–(c)). The area of the graph in Figure 4(b) significantly reduced compared to that in Figure 4(a), as in Figure 4(d). The number of potentiated channels is smaller than the number of activated channels. However, the graphical area of Figure 4(c) is significantly larger than that of Figure 4(a). In Figure 4(e), we can also see that the number of activated channels significantly increased (3.73 ± 0.70, *p* < .001, *n* = 15 slices/9 mice). These data indicate that forskolin induces the recruitment of silent responses in the ACC.

### NASPM blocked the recruitment of silent responses after forskolin-induced LTP in the ACC

**Figure 5.**
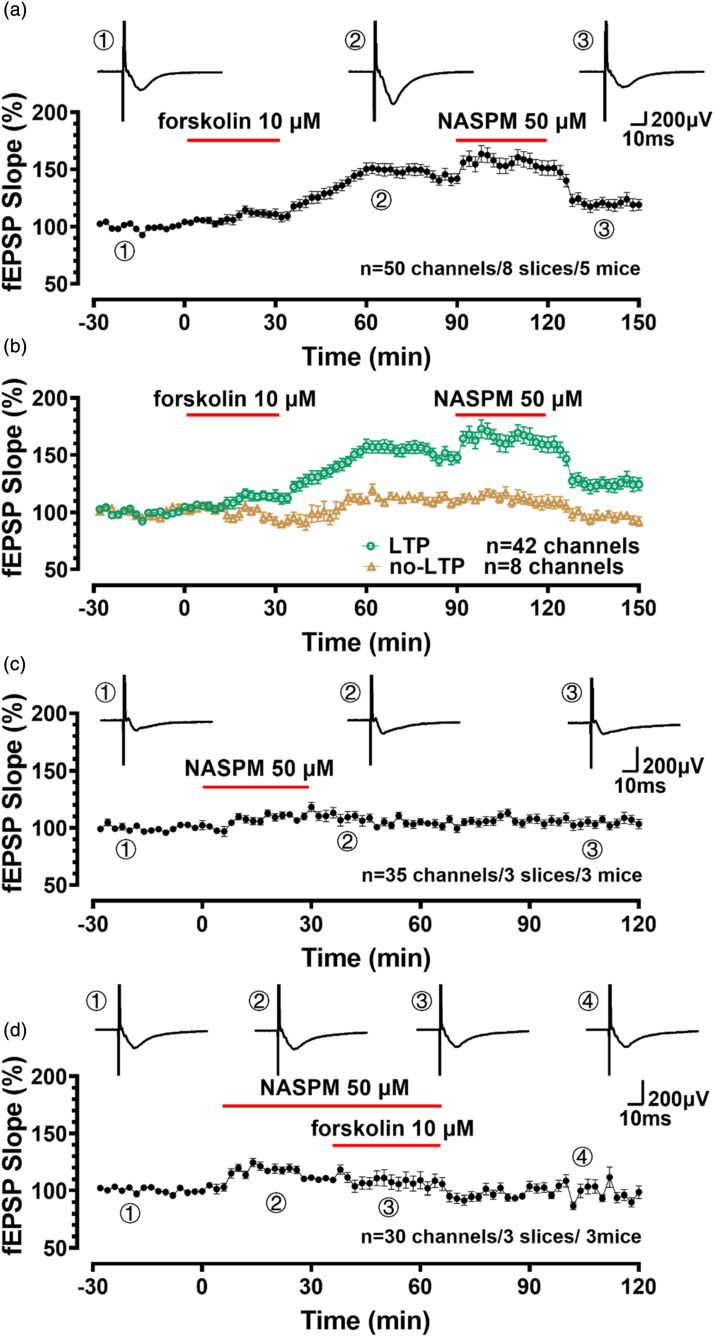
NASPM blocked the forskolin-induced LTP in the ACC (a) The averaged fEPSP slopes of bathing application of CP-AMPA receptor antagonist NASPM (50 μM) for 30 min reversed the LTP 1 h after forskolin application. The trace sample of baseline, forskolin-induced LTP and NASPM-reversed LTP are shown above. (b) The averaged fEPSP slopes of channels with forskolin-induced LTP and without LTP after perfusing NASPM for 30 min. (c) The fEPSP slope shows that bath application of NASPM for 30 min does not alter the basal transmission. (d) Bath application of NASPM blocked forskolin-induced LTP in the ACC.

The previous studies demonstrated that AMPA receptor-mediated eEPSCs were significantly inhibited by bath application of 1-naphthyl acetyl spermine (NASPM, 50 μM), one antagonist of CP-AMPA receptor, in mice after nerve injury.^25,26^ The fEPSP slope of forskolin-induced LTP was significantly reduced after the bath application of NASPM (154.5 ± 14.6% to 119.6 ± 14.3% of the baseline, *n* = 8 slices/5 mice, Figure 5(a)). In these slices, the fEPSP slope of potentiated channels reached 160.4 ± 6.4% of baseline after forskolin administration, and after bath application of NASPM, it reduced to 120.6 ± 4.5% of baseline (shown in Figure 5(b)). However, the fEPSP slope of no-potentiated channels does not change significantly (98.09 ± 1.6% of baseline, *n* = 35channels/3 slices/3 mice, Figure 5(b)). NASPM does not affect the slope of the baseline (101.3 ± 1.7% of baseline, Figure 5(c)), which is consistent with previous studies.^25^ Forskolin-induced LTP was blocked 30 min after the bath application of NASPM (107.3 ± 8.0% of baseline, *n* = 30 channels/3 slices/3 mice, Figure 5(d)).

**Figure 6.**
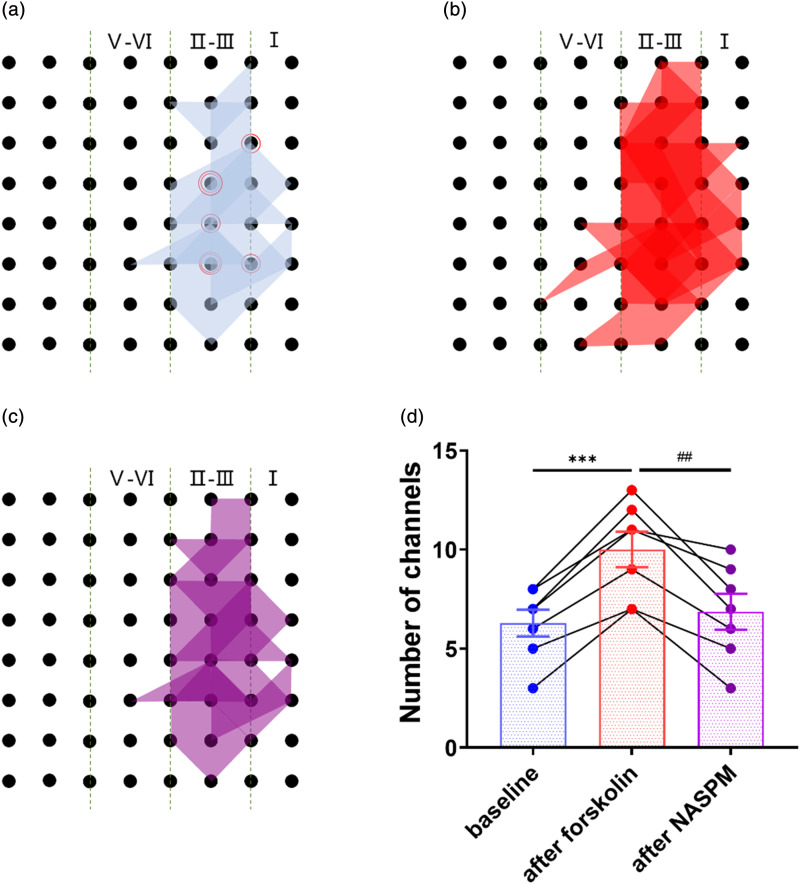
NASPM blocked recruited synaptic responses within the ACC after forskolin-induced LTP (a) The superimposed polygonal diagram shows the distribution of activated channels with fEPSP in the baseline state (blue). The red circle indicates the stimulation site (*n* = 7 slice/5 mice). (b) The red polygonal diagram shows the enlarged area of the activated channels compared to the baseline state after forskolin. (c) The superimposed polygonal diagram shows that activated channels (purple) have reduced after NASPM blocked forskolin-induced LTP. (d) The number of activated channels after the application of NASPM (50 μM) rescued recruited channels after the application of forskolin (10 μM) (F_(6, 12)_ = 16.92, one-way ANOVA, ***p < .001 for the number of baseline channels vs the number of channels after forskolin, ^**##**^ *p* < .01 for the number of channels after forskolin vs NASPM).

Interestingly, we further investigate whether NASPM affects the recruitment of silent channels. Figure 6(b) shows an increase in area compared to Figure 6(a), indicating that brain slices had the recruitment of silent channels. However, after the administration of NASPM, the number of recruited channels decreased, as shown in Figure 6(b) and (c). In Figure 6(d), the number of channels was significantly increased after forskolin compared to baseline (3.71 ± 0.42, ***p < .001, *n* = 7 slices/5 mice) and significantly decreased after NASPM compared to forskolin (3.14 ± 0.59, ^##^*p* < .01). The statistical result more clearly illustrates that the recruitment of silent channels was blocked by NASPM. These data indicate that forskolin-induced recruitment of silent responses requires CP-AMPA receptors.

## Discussion

In the present study, we provide strong evidence that silent responses exist in the cortical circuits within the ACC, and these silent responses can be recruited by activating ACs chemically. Our results suggest that ACs are likely to contribute to synaptic potentiation by at least two different mechanisms: (1) enhancing synaptic responses through existing active synapses; (2) increasing the recruitment of silent synapses. The effect of forskolin on cortical responses is input-specific; not all active responses were potentiated by forskolin. The recruitment of silent responses contributes to not only synaptic potentiation but also the spreading of LTP in the cortical circuit. Although the MED.64 recording system has its limitations, it allows us to evaluate silent responses at the circuit level of adult slices. Silent responses or synapses are difficult to study for whole-cell patch recordings since adult cortical pyramidal neurons are well-developed with many distal spines. Recent studies in the sensory cortex of adult animals have provided indirect and direct support for the existence of silent synapses in the adult cortex.^18,27^

**Figure 7.**
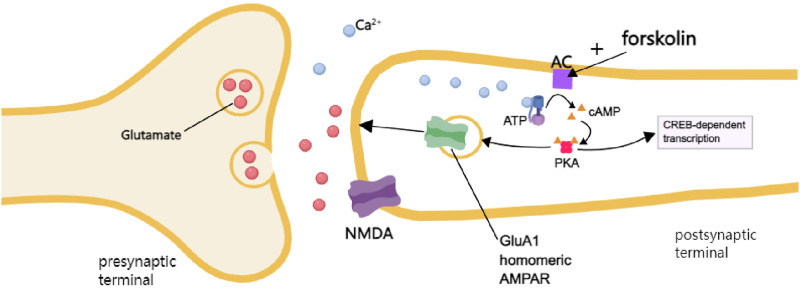
A signaling pathway of the recruitment of silent AMPA receptors after forskolin in the ACC A diagram showing the intracellular signaling pathway for the recruitment of silent synapses in adult synaptic spines. Forskolin induces the activation of ACs, and then leads to the enhancement of cAMP release. cAMP as a second messenger triggers PKA activity. The activation of PKA drives the insertion of CP-AMPA receptors (GluA1 homomeric AMPA receptors). Silent synapses transfer to functional synapses through the recruitment of CP-AMPA receptors.

In our previous studies, TBS-induced LTP in the ACC was able to recruit silent responses by the multi-channel recording.^19,20^ Furthermore, Song et al (2017) demonstrated that the phosphorylation of AMPA receptors contributes to not only LTP but also the recruitment of silent response.^10^ In the present study, we found that forskolin was able to induce the recruitment of silent responses, suggesting that activation of ACs is sufficient to recruit some silent responses (see Figure 7). This finding is consistent with previous studies using AC1 gene knockout mice or a selective inhibitor of AC1.^7^ Deletion of AC1 or inhibition of AC1 by NB001 inhibited ACC LTP and blocked the recruitment of silent responses in the ACC slices.^7,9^ Although we do not have evidence of activation of AC1 alone is sufficient, due to the lack of selective activator for AC1. Future studies are clearly needed when such an activator becomes available. In addition to LTP, previous studies have found that bath application of brain-derived neurotrophic factor (BDNF) and calcitonin gene-related peptide (CGRP) are able to recruit silent responses in the ACC of adult mice.^28–30^ Furthermore, neuronal subtype AC1 is required. These results suggest that cAMP pathways act downstream from BDNF and CGRP for the recruitment of silent responses. Similarly, Liu et al (2020) reported that CGRP produced synaptic potentiation and recruited silent responses in the insular cortex (IC), another cortical area that is important for the pain process.^31,32^

In a previous study using single-field electrode recording, Liauw et al (2005) reported that bath application of forskolin led to synaptic potentiation.^9^ However, it is unknown if forskolin may recruit silent responses in this study. In our study, using the MED64 recording system, we have confirmed that forskolin was able to induce synaptic potentiation in the ACC of adult mice. More interestingly, we found that forskolin did not potentiate all active synapses. In some channels, field synaptic responses were not potentiated by the bath application of forskolin. In parallel, using the same multiple recording system, we have consistently found that TBS does not produce synaptic potentiation in all active channels^7^; suggesting that these synapses are not homogenous in responding to LTP-induced stimuli or chemical application. Future studies are needed to investigate these differences among different channels.

In this work, the application of NASPM blocked not only the potentiation of synaptic plasticity but also the recruitment of silent responses induced by forskolin. It has been reported that NASPM is a selective inhibitor of GluA1-containing AMPA receptors.^33–35^ GluA1-containing AMPA receptors contribute to the recruitment of silent synapses in the ACC.^7,8^ The inhibition of forskolin-induced recruited responses by NASPM strongly suggests that GluA1-containing AMPA receptors are important for forskolin-produced effects. Song et al. (2017) found that the recruitment of TBS-induced silent responses was blocked in GluA1 845 knock-in mice, indicating that such recruitment is mediated by the phosphorylation of AMPA receptors at Ser 845 through cAMP-dependent PKA.^10^ We can infer that the recruitment of forskolin-induced and TBS-induced silent responses is likely to activate the same signaling pathway. Previous studies have demonstrated that NB001 blocked the recruitment of silent responses after TBS.^7,36^ Thus, NB001 may also inhibit the recruitment of chemically induced silent response. Future studies will be needed for investigating signaling pathways for the recruitment of silent responses.

In summary, our results demonstrate that forskolin induces LTP and the recruitment of silent responses in some, but not all the recording channels in the ACC. CP-AMPA receptors are significantly required in synaptic potentiation and the recruitment of silent responses. The significance of the LTP in the ACC has been well demonstrated.^1^ Our study further emphasized the importance of the cAMP-protein kinase A (PKA) signaling pathway in the LTP of the ACC neurons (Figure 7 for a model). Future experiments will be needed to explore the basic mechanism for regulation of these adult silent synapses, and their possible contribution to chronic pain and related emotional disorders. We believe that the MED64 system provides a useful research tool for future studies of adult silent synapses in different types of brain functions as well as brain diseases, and can be used for preclinical investigation of new drugs and medicine as well.
